# Diaqua­bis­(nitrato-κ*O*)bis­(pyridine-κ*N*)manganese(II)

**DOI:** 10.1107/S1600536812049161

**Published:** 2012-12-05

**Authors:** Naveed Alam, Muhammad Shahid, M. Khawar Rauf

**Affiliations:** a Department of Chemistry, Quaid-i-Azam University, Islamabad 45320, Pakistan

## Abstract

The structure of the title manganese complex, [Mn(NO_3_)_2_(C_5_H_5_N)_2_(H_2_O)_2_], consists of discrete monomeric entities with Mn^2+^ ions located on centres of inversion. The metal cation is octahedrally coordinated by a *trans*-N_2_O_4_ donor set with the pyridine N atoms located in the apical positions. Discrete mol­ecules are linked by O—H⋯O hydrogen bonds into one-dimensional supra­molecular infinite chains along the *b* and *c* axes.

## Related literature

For our previous work on the structural chemistry of transition metal complexes, see: Shahid *et al.* (2010 ▶). For details concerning the geometric parameters of Mn^II^ complexes, see: Saphu *et al.* (2012 ▶).
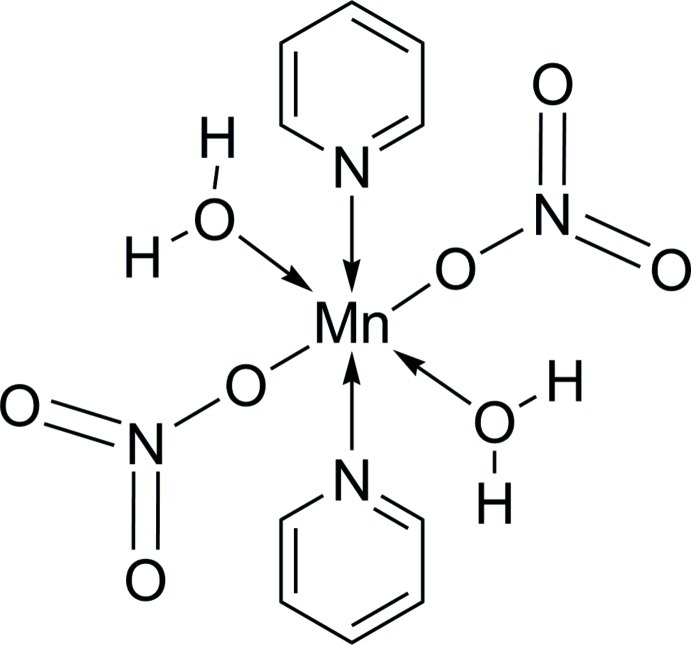


## Experimental

### 

#### Crystal data

[Mn(NO_3_)_2_(C_5_H_5_N)_2_(H_2_O)_2_]
*M* *_r_* = 373.19
Monoclinic, 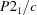

*a* = 8.8988 (7) Å
*b* = 11.8668 (10) Å
*c* = 7.5950 (6) Å
β = 107.500 (1)°
*V* = 764.91 (11) Å^3^
*Z* = 2
Mo *K*α radiation
μ = 0.91 mm^−1^
*T* = 100 K
0.43 × 0.39 × 0.39 mm

#### Data collection

Bruker SMART APEX CCD diffractometer
Absorption correction: multi-scan (*SADABS*; Bruker, 2003 ▶) *T*_min_ = 0.583, *T*_max_ = 0.701
6644 measured reflections
1897 independent reflections
1817 reflections with *I* > 2σ(*I*)
*R*_int_ = 0.016

#### Refinement

*R*[*F* ^2^ > 2σ(*F* ^2^)] = 0.023
*wR*(*F* ^2^) = 0.064
*S* = 1.08
1897 reflections
112 parameters
2 restraints
H atoms treated by a mixture of independent and constrained refinement
Δρ_max_ = 0.34 e Å^−3^
Δρ_min_ = −0.30 e Å^−3^

### 

Data collection: *SMART* (Bruker, 2002 ▶); cell refinement: *SAINT-Plus* (Bruker, 2002 ▶); data reduction: *SAINT-Plus*; program(s) used to solve structure: *SHELXTL* (Sheldrick, 2008 ▶); program(s) used to refine structure: *SHELXTL*; molecular graphics: *SHELXTL*; software used to prepare material for publication: *publCIF* (Westrip, 2010 ▶).

## Supplementary Material

Crystal structure: contains datablock(s) I, global. DOI: 10.1107/S1600536812049161/br2215sup1.cif

Structure factors: contains datablock(s) I. DOI: 10.1107/S1600536812049161/br2215Isup2.hkl

Additional supplementary materials:  crystallographic information; 3D view; checkCIF report

## Figures and Tables

**Table 1:** Hydrogen-bond geometry (Å, °)

*D*—H⋯*A*	*D*—H	H⋯*A*	*D*⋯*A*	*D*—H⋯*A*
O1—H1*B*⋯O4^i^	0.82 (1)	1.98 (1)	2.7805 (11)	163 (2)
O1—H1*A*⋯O2^ii^	0.84 (1)	2.63 (2)	3.2504 (11)	132 (1)
O1—H1*A*⋯O4^ii^	0.84 (1)	1.91 (1)	2.7495 (11)	174 (2)

Symmetry codes: (i) 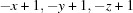; (ii) 
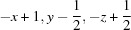
.

**Fig. 1.**
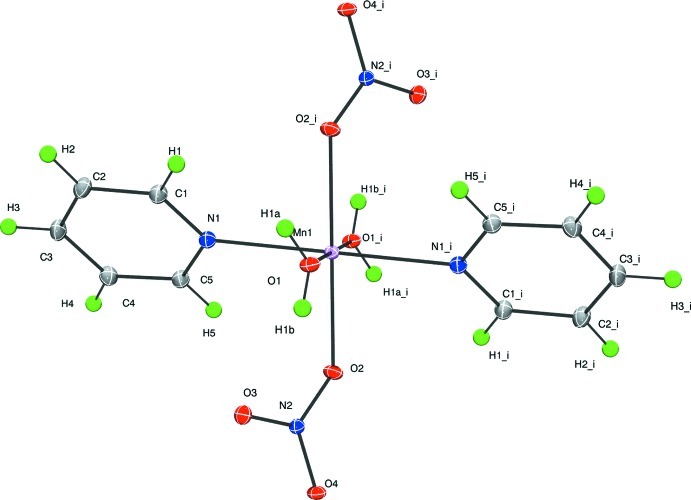
View of the molecular structure of the title compound with displacement ellipsoids drawn at the 50% probability level with atom labeling.

**Fig. 2.**
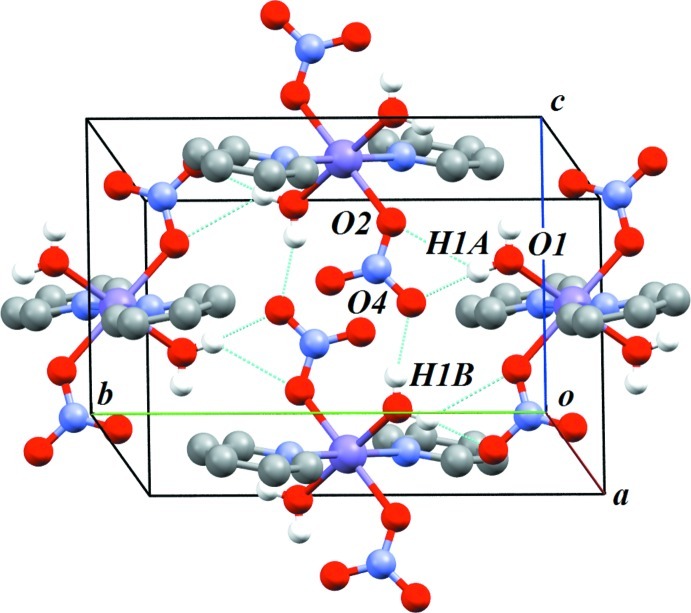
Packing diagram of the title compound. Hydrogen bonds are indicated by dashed lines.

## supplementary crystallographic information

### Comment

In relation to our previous work on the structural chemistry of transition metal complexes (Shahid *et al.*, 2010) as potential precursors for ceramic oxides of the type *M*O(*M* = Cu, Zn, Mn, Ni *etc*), the title compound was prepared as the unintented product of the recation of Mn(NO_3_)_2_.4H_2_O with potassium *O*-*n*-butyl xanthate in acetone and pyridine. The asymmetric unit of the title compound contains one pyridine, one nitrate and one water molecule coordinated with one Mn(II) atom. Fig. 1 shows a perspective view of the monomeric unit with the atomic numbering scheme. The Mn(II) atom is in a octahedral environment surrounded by two nitrate, two water and two pyridines ligands. As illustrated in Fig. 1, the Mn(II) atom is six-coordinated observing octahedral geometry with pyridine ligands located at apical positions. The Mn—O distance of nitrate are in good agreement with those reported in similar Mn^II^ complexes (Saphu *et al.*, 2012). In the crystal structure, molecules are assembled into one dimensional supramolecular infinite chains along *bc* axis through O—H···O intermolecular hydrogen bonds (Table1, Fig. 2).

### Experimental

Mn(NO_3_)_2_.4H_2_O (0.47 g, 0.27 mmol) was added to a stirred solution of potassium *O*-*n*-butyl xanthate(1.0 g,0.53 mmol) in acetone (30 ml). The contents were stirred until complete dissolution of the salt to which about 30 ml of pyridine was added and stirred for 1hr. Filtrate was kept under slow evaporation at room temperature to give the title compound as colourless crystals. Yield 60% (0.42 g), m.p. 373 K. Elemental analysis: calculated (found): C 32.18(31.78), H 3.78(3.35), N 15.01(15.35)%.

### Refinement

Water hydrogen atoms were tentatively found in the difference density Fourier map and were refined with an isotropic displacement parameter 1.5 that of the adjacent oxygen atom. The O—H distances were restrained to be 0.84Å within a standard deviation of 0.02 with U_iso_(H) =1.5 U_eq_(O). All other Hydrogen atoms were placed in calculated positions with C—H distances of 0.95Å for aromatic H atoms with U_iso_(H) =1.2 U_eq_(C).

### Crystal data

**Table:**

[Mn(NO_3_)_2_(C_5_H_5_N)_2_(H_2_O)_2_]	*F*(000) = 382
*M**_r_* = 373.19	*D*_x_ = 1.620 Mg m^−^^3^
Monoclinic, *P*2_1_/*c*	Melting point: 373 K
Hall symbol: -P 2ybc	Mo *K*α radiation, λ = 0.71073 Å
*a* = 8.8988 (7) Å	Cell parameters from 6452 reflections
*b* = 11.8668 (10) Å	θ = 2.4–30.5°
*c* = 7.5950 (6) Å	µ = 0.91 mm^−^^1^
β = 107.500 (1)°	*T* = 100 K
*V* = 764.91 (11) Å^3^	Block, colourless
*Z* = 2	0.43 × 0.39 × 0.39 mm

### Data collection

**Table:**

Bruker SMART APEX CCD diffractometer	1897 independent reflections
Radiation source: fine-focus sealed tube	1817 reflections with *I* > 2σ(*I*)
Graphite monochromator	*R*_int_ = 0.016
ω scans	θ_max_ = 28.3°, θ_min_ = 2.4°
Absorption correction: multi-scan (*SADABS* in *SAINT-Plus*; Bruker, 2003)	*h* = −11→11
*T*_min_ = 0.583, *T*_max_ = 0.701	*k* = −13→15
6644 measured reflections	*l* = −10→10

### Refinement

**Table:**

Refinement on *F*^2^	Primary atom site location: structure-invariant direct methods
Least-squares matrix: full	Secondary atom site location: difference Fourier map
*R*[*F*^2^ > 2σ(*F*^2^)] = 0.023	Hydrogen site location: inferred from neighbouring sites
*wR*(*F*^2^) = 0.064	H atoms treated by a mixture of independent and constrained refinement
*S* = 1.08	*w* = 1/[σ^2^(*F*_o_^2^) + (0.0364*P*)^2^ + 0.2526*P*] where *P* = (*F*_o_^2^ + 2*F*_c_^2^)/3
1897 reflections	(Δ/σ)_max_ < 0.001
112 parameters	Δρ_max_ = 0.34 e Å^−^^3^
2 restraints	Δρ_min_ = −0.30 e Å^−^^3^

### Special details

**Table:**

Geometry. All e.s.d.'s (except the e.s.d. in the dihedral angle between two l.s. planes) are estimated using the full covariance matrix. The cell e.s.d.'s are taken into account individually in the estimation of e.s.d.'s in distances, angles and torsion angles; correlations between e.s.d.'s in cell parameters are only used when they are defined by crystal symmetry. An approximate (isotropic) treatment of cell e.s.d.'s is used for estimating e.s.d.'s involving l.s. planes.
Refinement. Refinement of *F*^2^ against ALL reflections. The weighted *R*-factor *wR* and goodness of fit *S* are based on *F*^2^, conventional *R*-factors *R* are based on *F*, with *F* set to zero for negative *F*^2^. The threshold expression of *F*^2^ > σ(*F*^2^) is used only for calculating *R*-factors(gt) *etc*. and is not relevant to the choice of reflections for refinement. *R*-factors based on *F*^2^ are statistically about twice as large as those based on *F*, and *R*- factors based on ALL data will be even larger.

### Fractional atomic coordinates and isotropic or equivalent isotropic displacement parameters (Å^2^)

**Table:**

	*x*	*y*	*z*	*U*_iso_*/*U*_eq_	
C1	0.73516 (12)	0.28979 (9)	0.04145 (16)	0.0191 (2)	
H1	0.6397	0.2500	−0.0121	0.023*	
C2	0.87624 (13)	0.23034 (10)	0.08651 (17)	0.0225 (2)	
H2	0.8768	0.1517	0.0636	0.027*	
C3	1.01597 (13)	0.28769 (10)	0.16539 (17)	0.0217 (2)	
H3	1.1140	0.2491	0.1975	0.026*	
C4	1.01000 (13)	0.40250 (10)	0.19649 (16)	0.0209 (2)	
H4	1.1038	0.4440	0.2507	0.025*	
C5	0.86473 (13)	0.45545 (9)	0.14696 (15)	0.0185 (2)	
H5	0.8614	0.5342	0.1681	0.022*	
Mn1	0.5000	0.5000	0.0000	0.01221 (8)	
N1	0.72797 (10)	0.40102 (8)	0.07038 (12)	0.01619 (18)	
N2	0.62538 (9)	0.58526 (7)	0.40827 (11)	0.01339 (17)	
O1	0.38723 (9)	0.37524 (6)	0.12260 (10)	0.01650 (16)	
H1A	0.3806 (18)	0.3081 (12)	0.085 (2)	0.025*	
H1B	0.3892 (18)	0.3773 (14)	0.2317 (19)	0.025*	
O2	0.58830 (9)	0.61467 (7)	0.23972 (10)	0.01857 (17)	
O3	0.64860 (10)	0.48619 (6)	0.45430 (12)	0.01931 (17)	
O4	0.63944 (9)	0.66239 (6)	0.52668 (10)	0.01668 (16)	

### Atomic displacement parameters (Å^2^)

**Table:**

	*U*^11^	*U*^22^	*U*^33^	*U*^12^	*U*^13^	*U*^23^
C1	0.0167 (5)	0.0158 (5)	0.0234 (5)	−0.0013 (4)	0.0042 (4)	−0.0019 (4)
C2	0.0206 (5)	0.0153 (5)	0.0303 (6)	0.0011 (4)	0.0057 (4)	−0.0027 (4)
C3	0.0162 (5)	0.0206 (5)	0.0273 (6)	0.0029 (4)	0.0051 (4)	0.0010 (4)
C4	0.0161 (5)	0.0202 (5)	0.0242 (5)	−0.0024 (4)	0.0026 (4)	−0.0007 (4)
C5	0.0187 (5)	0.0141 (5)	0.0212 (5)	−0.0015 (4)	0.0039 (4)	−0.0013 (4)
Mn1	0.01386 (13)	0.01072 (13)	0.01179 (12)	−0.00029 (7)	0.00348 (9)	−0.00047 (7)
N1	0.0160 (4)	0.0152 (4)	0.0171 (4)	−0.0005 (3)	0.0046 (3)	−0.0003 (3)
N2	0.0134 (4)	0.0130 (4)	0.0136 (4)	−0.0001 (3)	0.0037 (3)	−0.0008 (3)
O1	0.0238 (4)	0.0131 (4)	0.0138 (3)	−0.0021 (3)	0.0075 (3)	−0.0013 (3)
O2	0.0277 (4)	0.0160 (4)	0.0107 (3)	−0.0003 (3)	0.0038 (3)	0.0001 (3)
O3	0.0250 (4)	0.0122 (4)	0.0199 (4)	0.0026 (3)	0.0055 (3)	0.0022 (3)
O4	0.0240 (4)	0.0134 (4)	0.0122 (3)	−0.0010 (3)	0.0049 (3)	−0.0025 (3)

### Geometric parameters (Å, º)

**Table:**

C1—N1	1.3428 (14)	Mn1—O1	2.1513 (8)
C1—C2	1.3902 (15)	Mn1—O1^i^	2.1514 (8)
C1—H1	0.9500	Mn1—O2^i^	2.2189 (7)
C2—C3	1.3856 (16)	Mn1—O2	2.2189 (7)
C2—H2	0.9500	Mn1—N1^i^	2.2646 (9)
C3—C4	1.3864 (16)	Mn1—N1	2.2646 (9)
C3—H3	0.9500	N2—O3	1.2262 (11)
C4—C5	1.3839 (15)	N2—O4	1.2627 (11)
C4—H4	0.9500	N2—O2	1.2710 (11)
C5—N1	1.3457 (13)	O1—H1A	0.843 (13)
C5—H5	0.9500	O1—H1B	0.824 (13)
			
N1—C1—C2	122.85 (10)	O2^i^—Mn1—O2	180.00 (3)
N1—C1—H1	118.6	O1—Mn1—N1^i^	87.58 (3)
C2—C1—H1	118.6	O1^i^—Mn1—N1^i^	92.42 (3)
C3—C2—C1	118.93 (10)	O2^i^—Mn1—N1^i^	93.06 (3)
C3—C2—H2	120.5	O2—Mn1—N1^i^	86.94 (3)
C1—C2—H2	120.5	O1—Mn1—N1	92.42 (3)
C2—C3—C4	118.74 (10)	O1^i^—Mn1—N1	87.58 (3)
C2—C3—H3	120.6	O2^i^—Mn1—N1	86.94 (3)
C4—C3—H3	120.6	O2—Mn1—N1	93.06 (3)
C5—C4—C3	118.71 (10)	N1^i^—Mn1—N1	180.0
C5—C4—H4	120.6	C1—N1—C5	117.46 (9)
C3—C4—H4	120.6	C1—N1—Mn1	123.68 (7)
N1—C5—C4	123.30 (10)	C5—N1—Mn1	118.85 (7)
N1—C5—H5	118.3	O3—N2—O4	121.32 (9)
C4—C5—H5	118.3	O3—N2—O2	121.39 (9)
O1—Mn1—O1^i^	180.0	O4—N2—O2	117.29 (8)
O1—Mn1—O2^i^	80.61 (3)	Mn1—O1—H1A	119.7 (11)
O1^i^—Mn1—O2^i^	99.39 (3)	Mn1—O1—H1B	122.9 (11)
O1—Mn1—O2	99.39 (3)	H1A—O1—H1B	110.5 (15)
O1^i^—Mn1—O2	80.61 (3)	N2—O2—Mn1	125.36 (6)

Symmetry code: (i) −*x*+1, −*y*+1, −*z*.

### Hydrogen-bond geometry (Å, º)

**Table:**

*D*—H···*A*	*D*—H	H···*A*	*D*···*A*	*D*—H···*A*
O1—H1*B*···O4^ii^	0.82 (1)	1.98 (1)	2.7805 (11)	163 (2)
O1—H1*A*···O2^iii^	0.84 (1)	2.63 (2)	3.2504 (11)	132 (1)
O1—H1*A*···O4^iii^	0.84 (1)	1.91 (1)	2.7495 (11)	174 (2)

Symmetry codes: (ii) −*x*+1, −*y*+1, −*z*+1; (iii) −*x*+1, *y*−1/2, −*z*+1/2.
